# Characterization of complex fluvial-deltaic deposits in Northeast India using Poisson impedance inversion and non-parametric statistical technique

**DOI:** 10.1038/s41598-022-21444-5

**Published:** 2022-10-08

**Authors:** M. Nagendra Babu, Venkatesh Ambati, Rajesh R. Nair

**Affiliations:** Computational Petroleum Geomechanics Laboratory, Department of Ocean Engineering, Indian Institute of Technology Madras, Chennai, India

**Keywords:** Solid Earth sciences, Geology, Geophysics

## Abstract

Characterizing complex fluvial-deltaic deposits is a challenging task for finding hydrocarbon discoveries. We described a methodology for predicting the hydrocarbon zones from complex well-log and prestack seismic data. In this current study, data analysis involves an integrated framework based on Simultaneous prestack seismic inversion (SPSI), target correlation coefficient analysis (TCCA), Poisson impedance inversion, and non-parametric statistical analysis, and Bayesian classification. First, seismic elastic attributes from prestack seismic data were estimated. They can provide the spatial distribution of petrophysical properties of seismic data. Then target correlation coefficient analysis (TCCA) was estimated roration factor “c” from well-log data. Using the seismic elastic attributes and rotation factor “c”, Poisson impedance inversion was performed to predict the Poisson impedance volume. Finally, Bayesian classification integrated the Poisson impedance volume with non-parametric probabilistic density functions (PDFs) to estimate the spatial distribution of lithofacies. Despite complex characteristics in the elastic properties, the current study successfully delineated the complex fluvial-details deposits. These results were verified with conventional findings through numerical analysis.

## Introduction

The lithological types and the hydrocarbon saturation zone are essential in the characterization of reservoir^1^. Modeling and characterization of lithology are critical in basin analysis and subsequent studies such as drilling and reservoir development studies^2^. The most difficult challenge in reservoir studies is obtaining accurate lithology and fluid saturation zones from various sources, particularly seismic data^3^. The most uncertainty is related to seismic information due to low resolution, non-unique solutions of seismic inversion techniques, and the relation between well-log and seismic data^4,5^.

The geoscientist's quest in the seismic interpretation defines the relationship between geophysical Data (Seismic data and well data) and reservoir properties to predict lithology distribution and mapping fluid-saturated zones^6^. Geoscientist's frequently used seismic elastic characteristics and rock physical constants to distinguish lithologies and locate hydrocarbon-saturated zones^7^. Seismic elastic attributes such as P-impedance (Z_P_), S-impedance (Z_S_), density (ρ), and V_P_/V_S_ ratio are used for lithology characterization by incorporating wireline log data^8^. Rock physical attributes such as Young modulus (E), shear modulus(u), bulk modulus(k), Lambda-rho (λ), and Mu-rho (µρ) have been used to identify hydrocarbon saturated zone^9^. Several methods have been proposed in quantitative seismic interpretation to estimate fluid-saturated zones and identify different rock types^10,11^. For a few years, seismic elastic properties have been used to discriminate hydrocarbon zone from other zones (Brine sand and shale)^12,13^. The seismic inversion technique (prestack inversion and AVO analysis) has become essential for understanding elastic properties (Z_P_, Z_S_, ρ & V_P_/V_S_ ratio) of lithology types and petrophysical parameters^14^. A study explained the practical approach by the different seismic inversion techniques to identify the lithofacies^15^. Another study explained lithofacies classification from seismic inversion in a geothermal reservoir^16^. Another researcher differentiated rock type based on petrophysical properties and estimated the permeability zones^17^.

**Figure 1:**
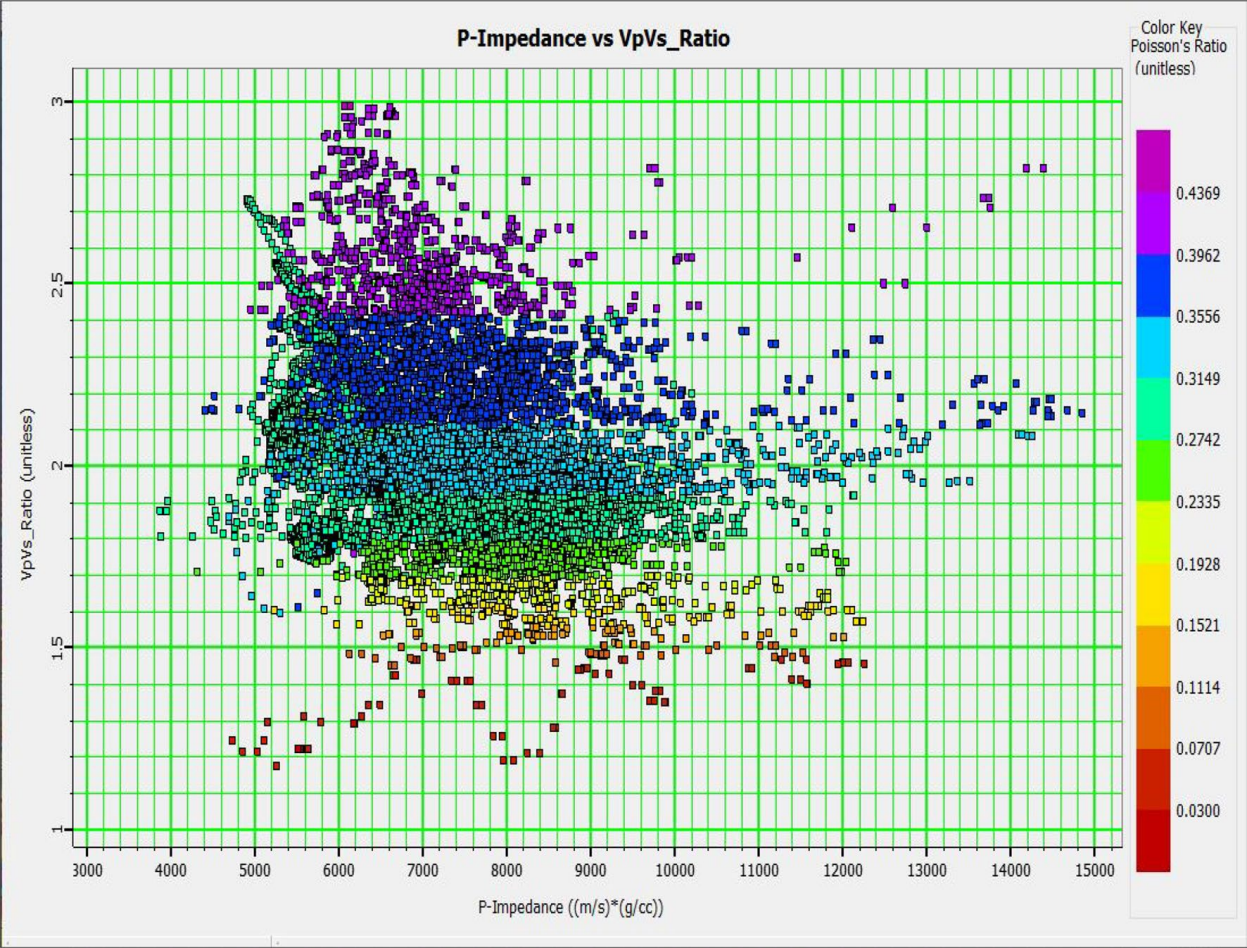
Cross plot of acoustic impedance (Z_P_) and V_P_/V_S_ ratio.

Characterizing the prospective zones from the other lithological zones in some reservoirs is very challenging if all have similar characteristics^18^. A study characterized the low resistivity low contrast reservoirs using the lithology impedance attribute^19^. Conventionally, low Poisson ratio and low-density values indicate hydrocarbon in many reservoirs, and those values are separated in a cross-plot if the lithology is clean sand C^20^. However, the sand quality might be different and not as clean in reality. Many difficulties are encountered when those elastic properties have similar properties, and characterizing lithologies can be complex^21^. Figure 1 shows the Z_P_ and V_P_/V_S_ ratio cross plot in the study area. Here observed, no proper separation of data points in the cross plot. As the color code of data points mentioned, the low Poisson ratio values belong to the hydrocarbon zone, but Z_P_ range values are almost the same for all zones. So only the Poisson ratio or V_P_/V_S_ ratio plays a significant role in the classification.

On the other hand, rock physical analysis can be applied to create the relation between rock properties (well log data) and seismic attributes^22^. Rock physics template (RPT) based on cross-plot analysis uses different rock properties to identify hydrocarbon saturated zone and lithology^23^. However, since an oil reservoir has indistinguishable elastic properties from lithologies and fluid-statured zones, conventional techniques such as prestack inversion, AVO analysis, and RPT are not enough to explicitly characterize brine hydrocarbon zones^24^.

A notable attribute named Poisson impedance (PI) was introduced to address the issues faced in those reservoirs^25^. The PI has defined as the difference between Z_P_ and scaled Z_S_. Poisson impedance helps characterize a reservoir with fluid content as an elastic constant. Poisson impedance inversion has given remarkable accomplishments in distinguishing different lithologies in the oil and gas industry^26^. The PI is used as a fluid factor in identifying the fluid content in the sandstone reservoir^27^. A scaled factor in the Poisson relation is crucial for Poisson impedance inversion success. A scale factor (c) is measured from the slope of the cross plot between compressional impedance (Z_P_) and shear impedance (Z_S_). An accurate measure of “c” is a critical task for the meaningful interpretation of PI^18^.

The Target correlation coefficient analysis (TCCA) was used in this study to estimate the accurate rotation parameter “c”^28^. The TCCA method has been used in many studies to estimate the “c” factor^29^. The hydrocarbon reservoir was characterized by Poisson impedance inversion, which used the TCCA to estimate factor “c”^30^. The TCCA can be applied to the GR, resistivity, and water saturation log. GR log can be used for lithofacies classification, and the resistivity log can be used for fluid classification through the fluid impedance^31^.

The non-parametric statistical classification based on the kernel density estimator was implied in this study to estimate the Probabilistic Density Functions (PDFs)^32^. The kernel density estimator of non-parametric statistical classification was suitable for geophysical data like well-log data^33^. A study has discussed lithology prediction using the borehole data using the non-parametric density estimator^34^. In many studies, kernel density estimators are efficiently distributed classes comparable to parametric methods^35^. The bandwidth (h) of the kernel operator is the single parameter to be determined in non-parametric kernel estimation. The kernel-based non-parametric statistical method provides smoother density functions^36^. Unlike the parametric method, these classification methods do not require predefined parameter assumptions/restrictions. As in the parametric method, assumptions of PDFs are complicated for geophysical data. So this non-parametric kernel estimator avoids the significant restrictions of the parametric approach^37^. Finally, the Bayesian approach was used in this study to estimate lithology volume for lithologies by combining the PDFs of non-parametric kernel estimator and seismic inputs (Poisson impedance & V_P_/V_S_ ratio). The Bayesian classification methodology is convenient for dealing with complex problems^38^. The Bayes' rule can integrate the different data sources and analyze the uncertainty^39^.

This study adopted a workflow to predict the hydrocarbon saturated zone of a sandstone reservoir of the Tipam formation from the Upper Assam basin, India. The study area has similar seismic velocities and density values for different lithologies such as shale, brine sand, and hydrocarbon sand. These issues make it challenging to characterize the accurate fluid and lithology, which was impossible in conventional interpretation techniques.

### Geological setting

The Assam-Arakan Basin is a petroleum-rich province in Northeast India, consisting of various tectonic-controlled basins^40^. The eastern Himalayas bounded the Basin in the North, Mikir hills in the southwest, and Naga hills at the southeastern boundary^41^. The Upper Assam basin contains the depositional system from the Eocene to Mio-Pleistocene. The essential litho-units are the Tipam sediments and Girujan clay of the Miocene age, Barail formation from the Oligocene age, and Sylhet Limestone and Kopili formation from the Eocene^42^. However, every stratigraphic horizon from Miocene has shown indications of hydrocarbon deposits. The crucial source rocks are the coal-shale unit of the Barial group from the Oligocene age, the shale of Koplili formation from Eocene, and Sylhet/Tura's formations of Paleocene. Girujan clays in the Assam Shelf on the northern side and Bokabil clays on the southern part act as major seals in the Upper Assam Basin^14^. Furthermore, many interbedded shale bands within the Oligocene formation also act as the local seals within the group.

The target reservoir is the Tipam formation contains the significant producible sediments belonging to the Miocene age, deposited in the fresh-brackish water ecosystems in the Assam Basin^43^. This formation has been subdivided into Upper, Middle, and Lower Tipam. The middle Tipam formation has the sand/shale alteration sequence, and the Lower Tipam formation consists of the Arenaceous sequence^44^. The upper Tipam contains an arenaceous sequence. Oil and as occur in Tipam sands with porosity ranging from 15 to 22%.

**Figure 2:**
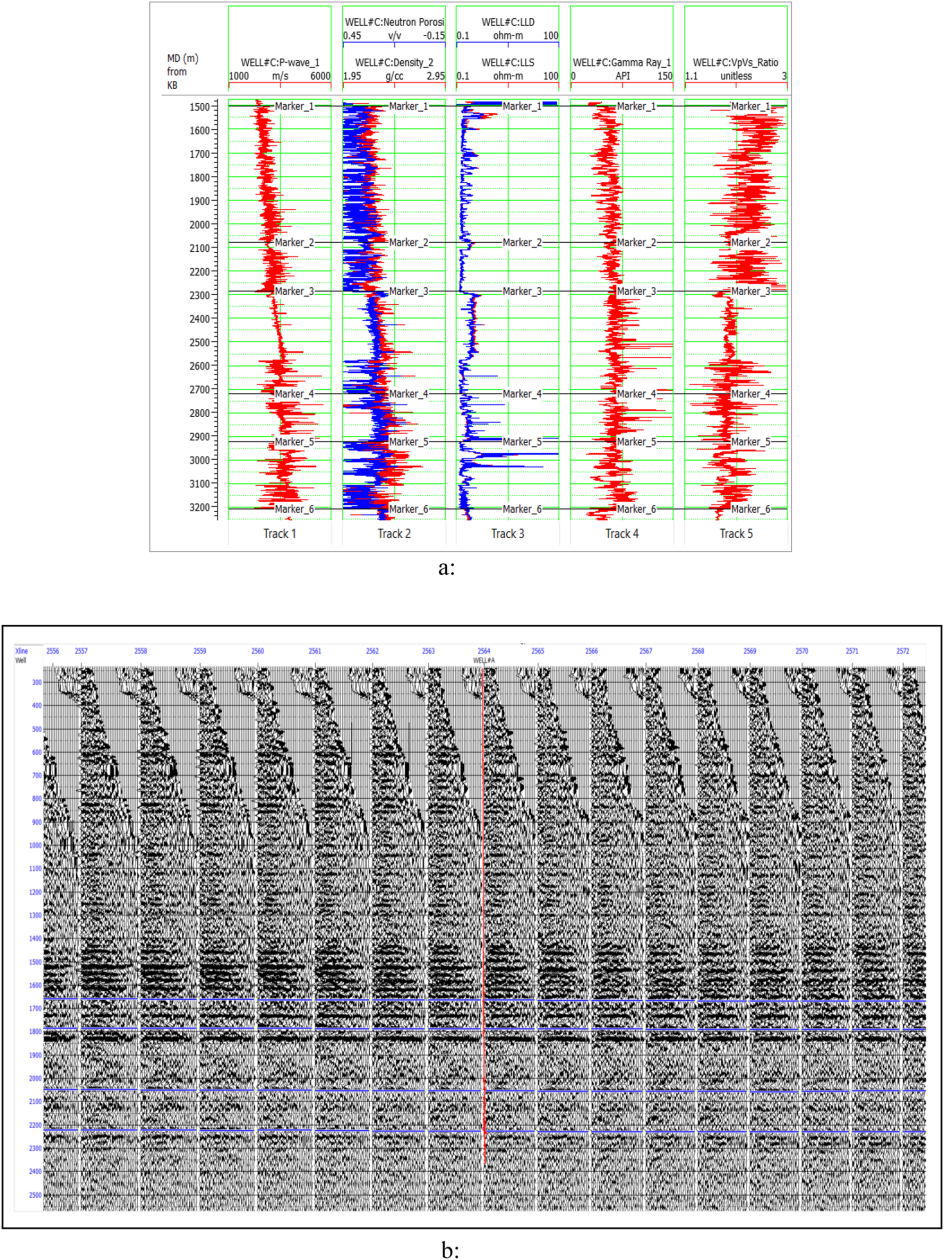
(**a**) Well logging curves of Well#03 in the study area. (**b**) Seismic Raw data (offset gathers) used in this study.

The data for analysis were borehole logs and prestack 3D seismic data volumes. The borehole logs have compressional slowness (DTCO), shear transit slowness (DTSM), density (ρ), resistivity logs (LLS, LLD & MSFL), and Gamma-ray (GR), Neutron porosity (NHPI) logs. After pre-conditioning, the seismic offset gathers have converted into angle gathers, which are required for prestack inversion. Well#A and Well#C was used for inversion and cross-plot analysis. Other well-logs are kept for quality check of inversion and classification results. Figure 2a shows available well-logging curves of Well#03, and Fig. 2b shows raw seismic offset gathers of the study area.

## Results and discussion

### Seismic attributes determination

**Figure 3:**
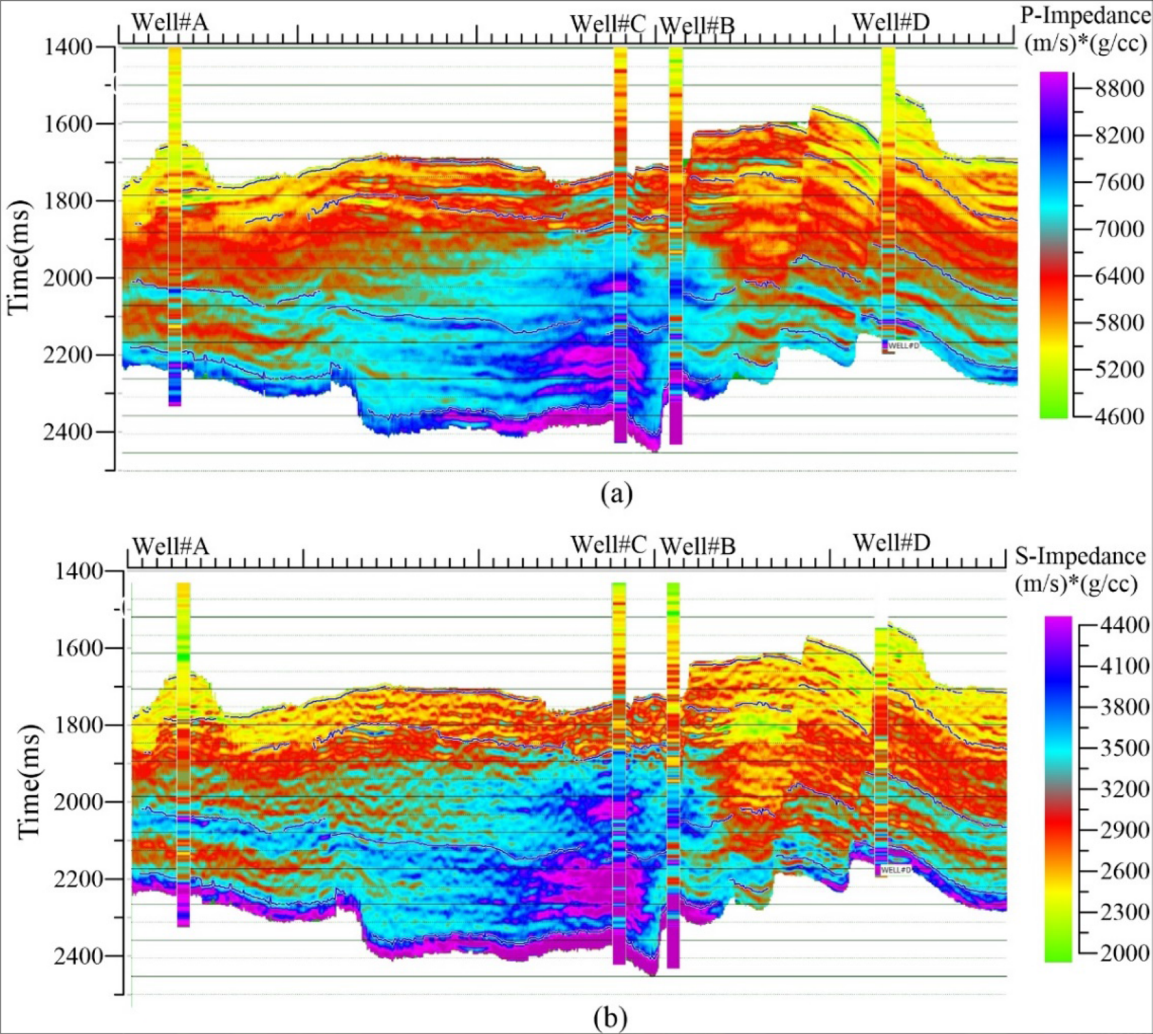
An arbitrary line of SPSI results along with well locations: (**a**) acoustic impedance, (**b**) shear impedance.

The SPSI technique was performed using the prestack seismic data and well-logs (Well#A & Well#C). We took advantage of SPSI in the generation of shear properties along with acoustic properties. This method transforms the prestack seismic data (angle gathers) into meaningful petrophysical parameters Z_P_, Z_S_, V_P_/V_S_ ratio, and density. The prestack inversion involves wavelet extraction (statistical and well based), well to seismic tie, estimation of the background model, and deterministic optimization using the simultaneous prestack inversion technique. Prestack angle gathers are inverted into elastic properties using well-based angle-dependent wavelets and an initial low-frequency model. Figure 3 shows an arbitrary line of inverted seismic volumes (Z_P_ & Z_S_) intersecting all wells, and inverted results were verified with inserted well properties (Z_P_ & Z_S_) in the seismic data.

As mentioned earlier, conventional interpretation attributes such as Z_P_ & V_P_/V_S_ ratio are used to classify the lithofacies. As seen in Table 1, conventional attribute Z_P_ is almost similar for classified lithofacies. As mentioned earlier, only V_P_/V_S_ ratio influences lithofacies characterization. This depositional complexity is difficult to classify with conventional attributes.

**Table 1:** Interpreted ranges of conventional attributes for lithofacies classification.

Lithofacies class	P-Impedance (Z_P_) ((m/s) (g/cc))	V_P_/V_S_ ratio
HC	3800–12,400	1.15–1.82
SAND	3500–14,900	1.82–2.12
SHALE	3500–13,900	2.12–3.1

### Poisson impedance (PI) volume extraction

**Figure 4:**
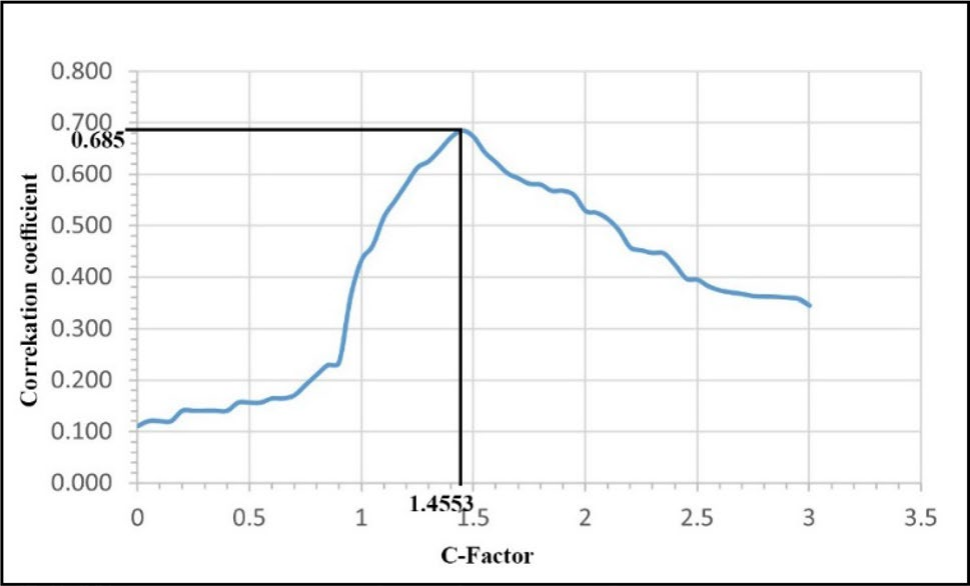
Rotation factor “c” estimation from the Target correlation coefficient analysis.

PI analysis was conducted as the second step of the methodology to estimate PI attribute volume from seismic elastic attributes and PI curves from well log data. An accurate value of rotation factor “c” is required to estimate the Poisson impedance volume, as mentioned in Eq. (2 in the material and methodology section. This rotation factor is generally obtained from regression analysis on the cross-plot of Z_P_ and Z_S_. However, TCCA was utilized to predict the accurate value of the rotation factor “c”. the correlation analysis was conducted using the GR log and the PI curves obtained by different “c” values. This correlation analysis shows a maximum correlation coefficient at the c-value of 1.4553 for the GR curve (cc = 0.685). This “c” value can be used to estimate Poisson impedance volume. Figure 4 shows the target correlation coefficient analysis to estimate the “c” value.

**Figure 5:**
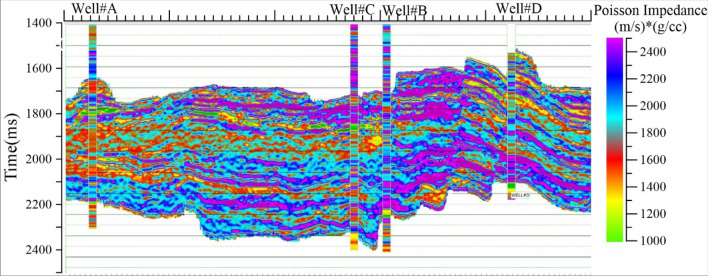
Arbitrary line of Poisson Impedance (PI) volume intersects at wellbore locations with PI property.

**Figure 6:**
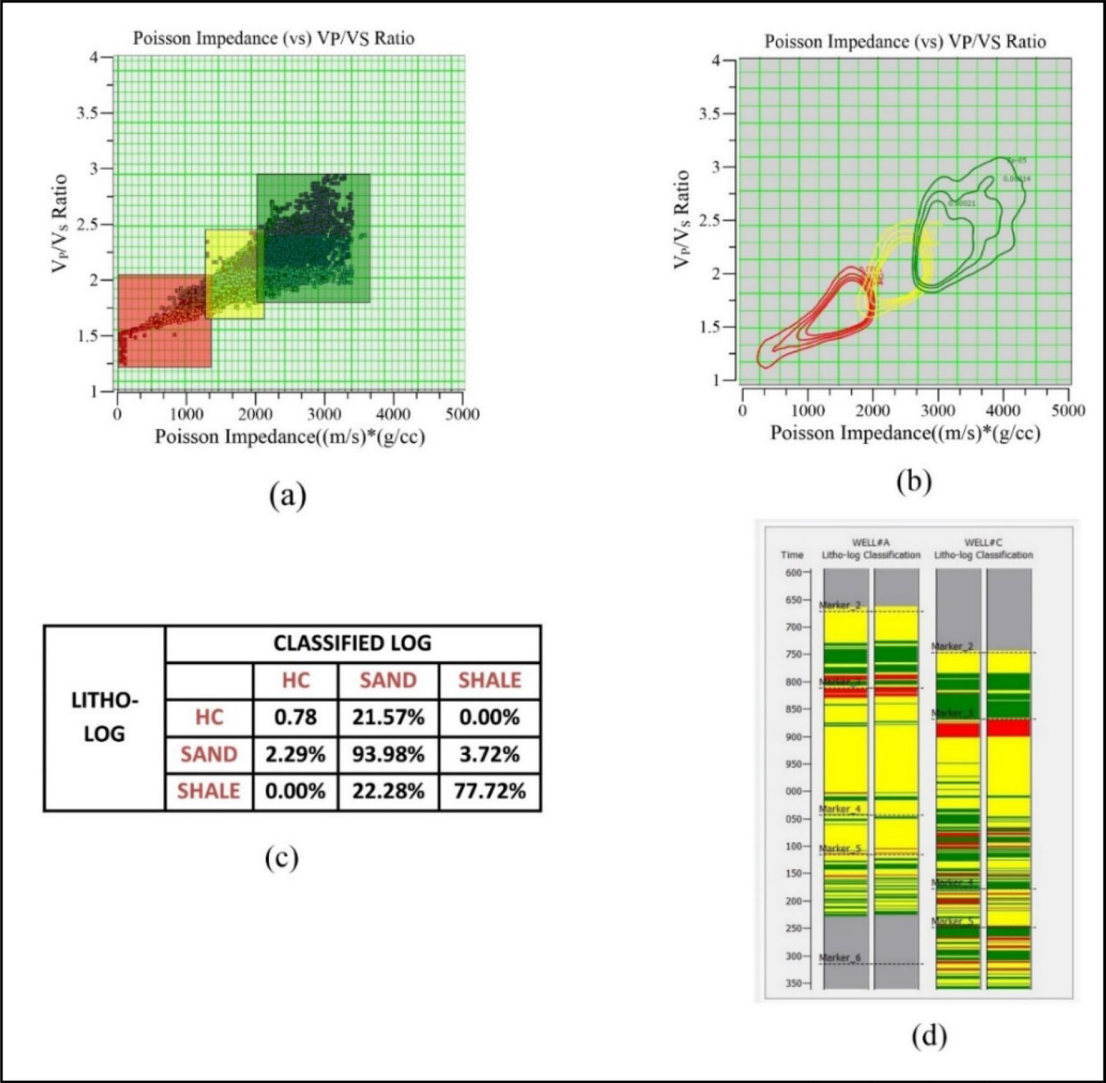
Lithofacies classification procedure and quality measures: (**a**) cross-plot analysis between PI and V_P_/V_S_ ratio; (**b**) non-parametric PDFs; (**c**) confusion matrix; (**d**) visual inspection of predicted lithofacies at wells (Well#A and Well#C).

As mentioned^25^ in Eq. (2), Poisson impedance inversion was performed using the estimated rotation factor “c” and seismic elastic volumes such as AI and SI. The PI inversion was applied to seismic volumes and borehole logs. The PI volume and curves were created using the Z_P_, Z_S_, and “c” factor (1.4553). The Arbitrary PI volume from seismic attributes is shown in Fig. 5. Figure 5 shows PI attributes volume that reveals that the hydrocarbon zone was observed to have a well-defined separation with the effectiveness of the PI attribute. Low PI values ranging from 500 ((m/s) *(g/cc) to 1450 ((m/s) *(g/cc) indicates the hydrocarbon zone as classified in cross plot analysis (Fig. 6a). PI values 1450–2100 (m/s) *(g/cc) indicates water-bearing sand lithology. PI values ranging from 2100–3200 (m/s) *(g/cc) corresponding to shale.

### Lithological characterization by non-parametric statistical technique and Bayesian classification

This section determines the spatial distribution of lithofacies by applying Bayesian classification using the non-parametric PDFs and Poisson impedance attribute. Mathematical information is provided in the methodology section. First, cross-plot analyses are generally conducted to separate different lithologies using seismic and well-log data^45^. The relation of different attributes is visually represented by cross plots for interpreting the hydrocarbon's presence and other lithologies. Different attribute pairs can be used to identify the lithologies by making clusters on data points of cross-plot^46^. The cross-plot analysis was performed using the PI curves and & V_P_/V_S_ ratio of Well#A and Well#C to characterize various lithofacies. Figure 6a shows cross-plot analysis, which was characterized as hydrocarbon-bearing zones with red color, sand with yellow, and shale with green. Table 2 shows PI & V_P_/V_S_ ratio values to characterize the different lithologies.

**Table 2:** Lithofaices characterized values of Poisson impedance (PI) and V_P_/V_S_ ratio.

Lithofacies class	Poisson impedance (PI) ((m/s) (g/cc))	V_P_/V_S_ ratio
HC	500–1450	1.15–1.82
SAND	1430–2100	1.60–2.45
SHALE	2050–3200	1.78–3.1

The non-parametric statistical mechanism was performed on the cross-plot data points (Fig. 6a) to estimate PDFs for each lithofacies. As mentioned in the methodology section, there is no parameter estimation in non-parametric statistical techniques for predicting PDFs. Based on the non-parametric methodology, different bandwidth (h) tried to estimate the PDFs and lithofacies prediction results verified with confusion matrix at well locations (Well#A and Well#B). The confusion matrix was applied between true lithofacies (from Well logs) and predicted logs (seismic lithofacies at well locations). Seismic lithofacies are estimated by integrating the seismic attributes and PDFs using Bayes' rule^1^. Here we finalized bandwidth as 4.93, which provided a better confusion matrix. The non-parametric PDFs for three lithofacies are shown in Fig. 6b.

**Figure 7:**
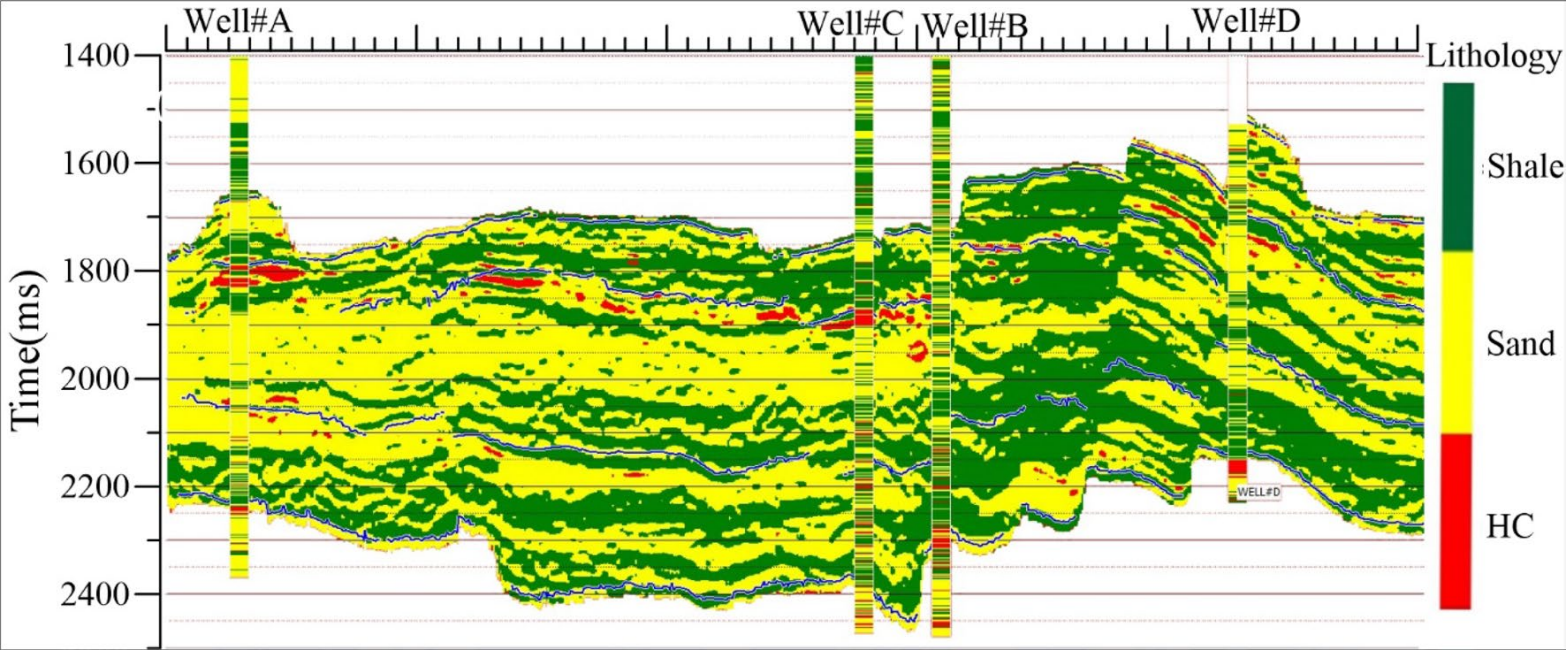
Arbitrary line of lithofacies model intersects at wellbore locations with inserting the true litho-logs.

The confusion matrix and visual comparison between the true and predicted lithofacies of Well #A and Well#C are shown in Fig. 6c,d. The column data in the confusion matrix was true data, and the row data represented predicted data. The more significant percentages in the confusion matrix indicate the quality of the results if the high values indicate a good match between the true and predicted lithofacies. It provides mismatch information between true lithofacies and predicted lithofacies at well locations. Figure 6d visually inspects predicted lithofacies of Well#A and Well#C with corresponding true lithofacies. Using the non-parametric PDFs, litho-logs (estimated from cross-plot classification), and seismic input such as Poisson impedance & V_P_/V_S_ ratio to estimate the lithofacies model by Bayesian classification. Lithologs help as prior information in Eq. 4^47^. Figure 7 shows the arbitrary line of lithofacies volume with three lithofacies such as hydrocarbon zone (Red color), sand (yellow), and shale (Green). intersecting at all well locations.

**Figure 8:**
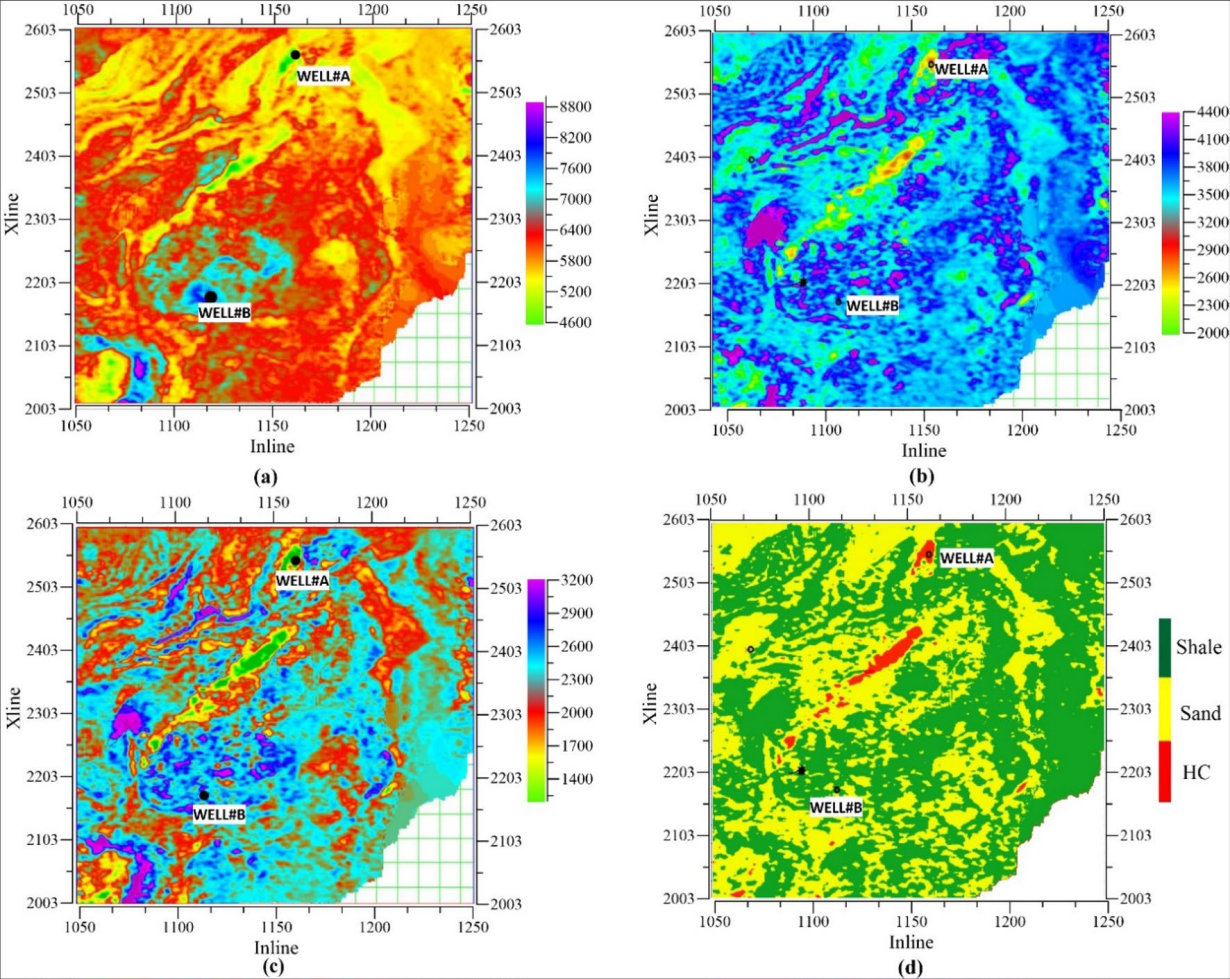
Horizontal slice of seismic attributes (**a**) acoustic impedance (**b**) shear impedance (**c**) Poisson impedance (**d**) Lithofaices at 1820 ms.

In this study, the estimated Poisson impedance attribute helped to classify the lithofacies. As seen in Table 2, two attributes (PI and V_P_/V_S_ ratio) play an important role in classifying lithofacies (Fig. 6a). Low PI (500–1450 ) ((m/s) *(g/cc) & low V_P_/V_S_ ratio values (1.15–1.82) were identified as hydrocarbon saturated zone, marked as red color in the lithology model (Fig. 7). The brine sand was differentiated by PI values ranging from 1450 (m/s) *(g/cc) to 2100 (m/s) *(g/cc) & V_P_/V_S_ ratio ranging from 1.60–2.45. The shale was modeled with values of PI 2100–3200 (m/s) *(g/cc) & V_P_/V_S_ ratio 1.78–2.43. Figure 8a,b shows three-dimensional slices of conventional attributes (Z_P_ and V_P_/V_S_ ratio) at 1820 ms in the seismic data. It was observed in Fig. 8a that acoustic values could not able characterize lithofacies due to almost similar values. However, PI impedance volume in Fig. 8c has clear separation values for different lithofacies, especially hydrocarbon zones. Figure 8d shows the horizontal slice of resultant lithofacies after applying Bayesian classification using the non-parametric PDFs, PI volume (Fig. 8c), and V_P_/V_S_ ratio (Fig. 8b,c). Hydrocarbon zones are characterized by red color, sand was in yellow color, and shale identified with green color. Comparison of 8a, 8b & 8d, PI attribute was clearly distinctive & having very low values than conventional attribute (Z_P_). Hence, the proposed framework successfully revealed HC, sand, and shale using the PI inversion and non-parametric statistical technique.

### Numerical analysis

**Figure 9:**
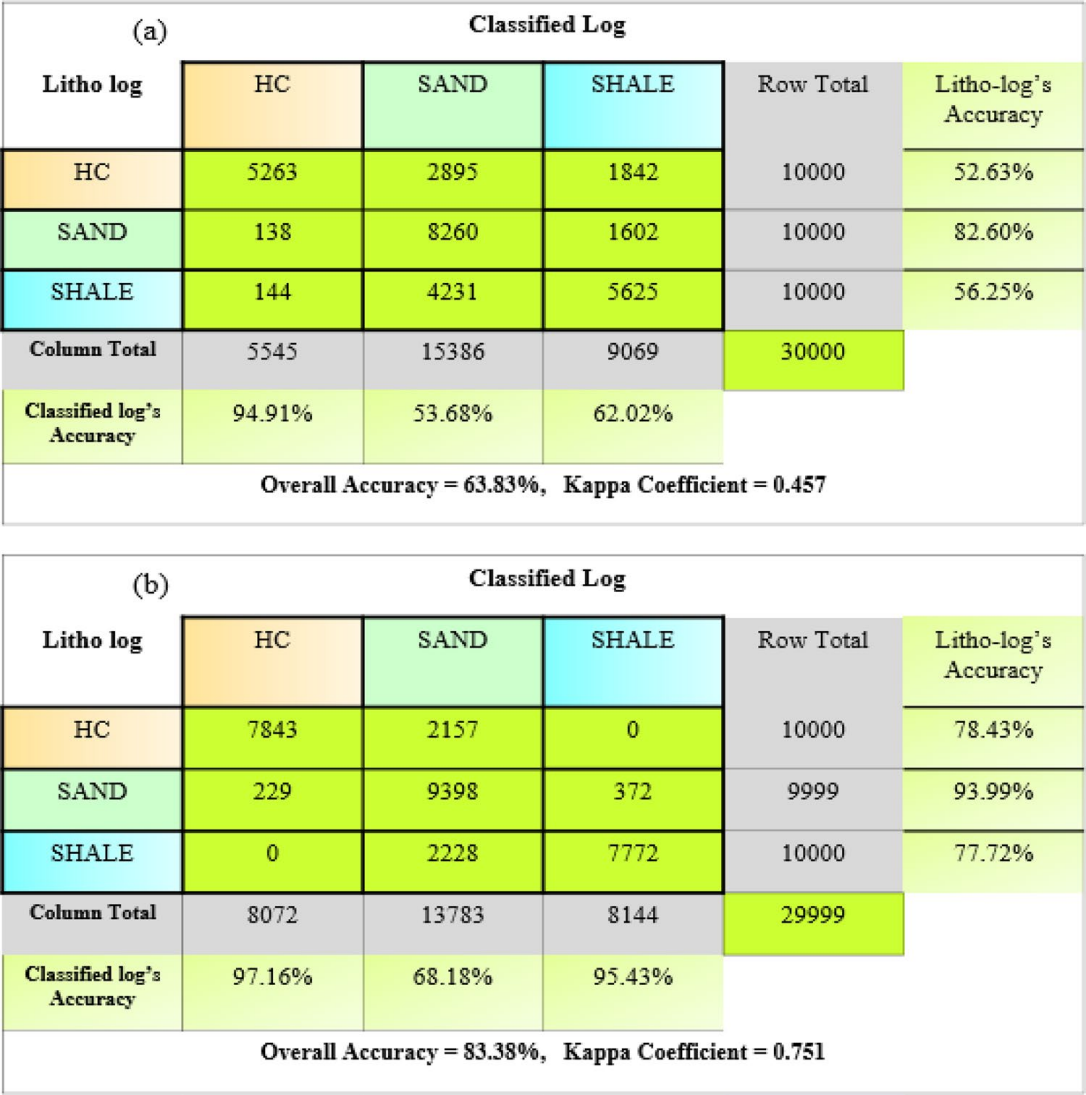
Confusion matrix Kappa coefficient and overall accuracy (**a**) conventional methodology (**b**) present methodology.

The superiority of the present methodology has been analyzed by comparing the results of non-parametric statistical classification from the different seismic inputs such as Z_P_ and PI volume generated from the traditional method, which involved an inverse slope of the cross plot of Z_P_ and Z_S_ in regression analysis to estimate rotation factor “c”. We have compared these results through the confusion matrix and kappa coefficient. The conventional interpretation results of the non-parametric statistical classification using the Z_P_ in this study area were taken from^48^. We evaluated the impact of these inputs on the results by comparing each prediction log with the true litho log. Figure 9a,b show the confusion matrix and kappa coefficient of these methodologies. The kappa coefficient determines the match between the prediction values and true values.

### Kappa coefficient

$$\hat{K} = \frac{{M\mathop \sum \nolimits_{i = j = 1}^{r} n_{ij} - \mathop \sum \nolimits_{i = j = 1}^{r} n_{i} *n_{j} }}{{M^{2} - \mathop \sum \nolimits_{i = j = 1}^{r} n_{i} *n_{j} }}$$

where *r* = number of rows in the confusion matrix, n_*ij*_ = number of observations in row *i*, column *j*, n_*i*_ = total number of observations in row *i*, n_*j*_ = total number of observations in row *j*, *M* = total number of observations in the matrix.

As observed 9a & b, overall accuracy is higher than the present methodology. It shows the overall accuracy is 83.38%, but the conventional methodology's overall accuracy was only 63.83, as shown in Fig. 9b. The adopted methodology predicted lithofacies better than the conventional methodology in complex fluvial-deltaic deposits from the Kappa coefficient score. Conventional attributes may be ineffective in distinguishing fluid-saturated zones from non-fluid-saturated zones for complex deposits.

## Conclusion

We applied an integrated framework to delineate the lithofacies of complex fluvial-deltaic sandstone reservoirs in an oilfield in Northeast India. Our outcomes proved that the adopted methodology improved the lithofacies distribution in the Upper Assam Basin. Additionally, our findings proved that PI was more effective in estimating hydrocarbon zone than conventional attributes. Seismic elastic attributes are estimated from prestack seismic data using the SPSI algorithm. An intermediate procedure of this methodology estimated the rotation factor “c” from the Target correlation coefficient analysis (TCCA) of well-logs. The PI volume and curve were estimated using the rotation factor “c”. Furthermore, the non-parametric statistical technique was applied to estimate PDFs for HC, sand, and shale. Finally, the lithofacies distribution was generated using PDFs, PI volume, and V_P_/V_S_ ratio in Bayesian classification. Our findings correspond to three lithofacies are characterized as HC with PI values (500–1450 ) ((m/s) *(g/cc) & low V_P_/V_S_ ratio values (1.15–1.82), sand deposits identified with PI values ranging from 1430 (m/s) *(g/cc) to 2100 (m/s) *(g/cc) & V_P_/V_S_ ratio ranging from 1.60–2.45. The shale was characterized modeled with values of PI 2050–3200 (m/s) *(g/cc) & V_P_/V_S_ ratio 1.78–3.1. The results efficiency was verified with numerical analysis through kappa coefficient analysis between conventional results and proposed framework results. This analysis has proven that the adopted methodology provided better lithofacies characterization (Annexure I).

## Materials and methodology

The methodology involves simultaneous prestack inversion, Poisson impedance analysis, and non-parametric statistical classification for explicitly identifying the hydrocarbon zone. The seismic prestack inversion technique was applied to predict the seismic elastic properties. Later, Poisson impedance analysis and target correlation coefficient analysis are applied to estimate the Poisson impedance from seismic and well log data. After that, cross plot analysis was conducted to identify different lithologies using the Poisson impedance and VP/VS curves. Finally, non-parametric statistical classification was used to estimate the probability density function. The Bayesian classification method is applied to the model distribution of hydrocarbon zones using Poisson impedance volume.

### Simultaneous prestack inversion (SPSI)

Seismic subsurface elastic properties such as Z_P_, Z_S_, ρ, and V_P_/V_S_ ratio are estimated from prestack seismic angle gathers using the simultaneous prestack seismic inversion technique^49^. Conventionally, the outcomes of prestack inversion can be used to optimize, identify prospects, and identify 'sweet spots' in field development studies. The prestack inversion procedure began by conditioning the seismic offset gathers to improve the signal-to-noise ratio by creating the super gather. This strategy reduces random noise while preserving the amplitude versus offset relationships. Using the seismic velocity field, this super gather in the offset domain was transformed to angle-gather for the angles between 0°–45°.

After preparation of angle gather, the well-seismic tie was performed to estimate time to depth relation for depth stratigraphic markers of well log data and time stratigraphic markers of seismic data using the angle-dependent wavelet. Two wavelet extraction methods, such as statistical and well-based, were used to estimate wavelets. First, the statistical method based on the autocorrelation concept was applied to estimate angle-dependent wavelets. These statistical wavelets were convolved with reflectivity from well-log data for synthetic seismic data. This synthetic seismic data is correlated with actual seismic data with a good correlation coefficient at all well locations. Following the acceptable correlation, well-based wavelets are estimated by designing a time-domain operator that is convolved with the actual seismic data. Prestack seismic inversion technique is a process to convert the seismic reflection data into a quantitative depiction of reservoir properties^50^. Simultaneous prestack inversion was explained to estimate the seismic elastic properties^51^. Study^52^ performed prestack inversion from the modified reflectivity equation^53^.

1 $${\text{RPP}}\left( {\uptheta } \right) = {\text{ A Rp}} + {\text{BRs}} + {\text{CRd}}$$

where R_PP_ (θ) is reflectivity, A = (1 + tan^2^ θ); B =  − 8(V_P_/V_S_)^2^ sin^2^ θ; C =  − 0.5tan^2^ θ + 2(V_P_/V_S_)^2^ sin^2^ θ, R_P_ = P Reflectivity = $\frac{1}{2 }\left[ {\frac{\Delta VP}{{VP}} + \frac{\Delta \rho }{\rho }} \right] = \frac{\Delta ZP}{{2ZP}}$, R_S_ = S − Reflectivity = $\frac{1}{2 }\left[ {\frac{\Delta Vs}{{Vs}} + \frac{\Delta \rho }{\rho }} \right] = \frac{\Delta Zs}{{2Zs}}$, Rd = density Reflectivity = $\frac{\Delta \rho }{\rho }$.

### Poisson impedance (PI) analysis

Poisson impedance analysis was proposed by^25^, which involved rotating the cross plot of P-impedance (Z_P_) and S-impedance (Z_S_) for classifying the hydrocarbon zone accurately from other lithofacies. This new parameter called Poisson impedance help as a rock physical parameter in the lithofacies classification. According to^54^, Poisson impedance is similar to the Fluid factor attribute. One particular rotation of the axis of the AI-SI cross-plot precisely distinguishes different lithologies and fluid zones. The PI attribute can estimate using a rotation that links the Poisson's ratio (σ) and density (ρ). The density (ρ) and Poison's ratio are significant parameters in the reservoir characterization for their low values for hydrocarbon saturated zones. The mathematical notation of the PI attribute is shown in the following equation as explaining a rotation of the AI-SI cross plot to discretize the lithologies.

2 $${\text{PI}} = {\text{ AI}} - {\text{c SI}}$$

where “c” is a rotation parameter, AI is P-Impedance (Z_P_)/Acoustic impedance, SI is Shear impedance (Z_S_).

The rotation factor “c” is critical in computing Poisson impedance (PI). The rotation parameter “c” is generally determined using a regression analysis of the AI and SI cross plot for the wet trend^18^. However, it will not always provide accurate value due to fitting issues in regression analysis and is also highly influenced by log quality. Another approach, Target correlation coefficient analysis (TCCA), was another approach to obtaining accurate rotation factor “c”. The mathematical notation of the TCCA is explained by^28^. Generally, different logging curves such as Gamma-ray, water saturation, porosity, resistivity, etc., are used to estimate the rotation factor. In this study, the GR log calculated the “c” factor to estimate the Poisson impedance^55^. As introduced^29^, the correlation coefficients between PI curve with different c-values and GR log.

### Non-parametric statistical classification technique & Bayesian modeling

The parametric and non-parametric statistical methods are essential for estimating probability densities. The first requires many assumptions with known PDFs with predefined parameters such as mean value (μ) and deviation (s). The non-parametric statistical classification does not require predefined restrictions as conventional parametric statistical classification. Another advantage is that it uses the data directly without estimating theoretical parameter distribution^37^. So there is no error and mismatch between the estimated and actual trend of lithology distribution.

This study used non-parametric statistical classification to estimate probabilistic density functions on the lithofacies cross plot between Poisson impedance and V_P_/V_S_ ratio. It was used to avoid the assumptions that are required in the parametric approach. The kernel estimator of the non-parametric classification method was applied to estimate the smother PDFs from the cross-plot space of Poisson impedance and V_P_/V_S_ ratio^56^. The basic notation to analyze univariate data points is the probability density function for non-parametric data distribution^57^. The density function equation for a random variable that included the probability density function f(x) is as follows

$$P\left( {a < X < b} \right) = \mathop \smallint \limits_{a}^{b} f\left( x \right)dx$$

For any constants a and b.

By using this density function definition, the probability density function can be constructed. There are two probability density estimators: histogram and smooth density estimator. The kernel density estimation is a simple expanded histogram method. However, the histogram method is discrete and does not provide smooth density functions. In the smooth density estimator, summing all kernel functions in the data provides a smooth representation of the PDFs. The probability density function can be estimated using a non-parametric kernel estimator defined as the following equation for n variable data points (X_1_, X_2_,…., X_n_) in the cross plot^58^.

3 $$fn\left( x \right) = \frac{1}{nh}\mathop \sum \limits_{i = 1}^{n} \left( K \right)\left( {\frac{x - Xi}{h}} \right)$$

where K is a kernel function, h is smoother operator length, and sample size indicates by n (X_1_, X_2_,…, X_n_).

The smoothing parameter, or bandwidth, h, determines the degree to which the data are smoothed. Minimizing the mean square error yields the optimal bandwidth value^57^. The critical objective is to select an appropriate operator bandwidth (h). Kernel functions that employ Gaussian functions are quite frequent^59^. The Epanechnikov kernel was utilized in this investigation because it had an advantage over Gaussian functions in that it was zero outside of its range. So it has a finite length and is optimal for the minimum variance. Numerous studies discussed that there is no objective technique for determining the optimum bandwidth (h). However, there is a challenge in estimating accurate density function from non-parametric statistical classification, especially in high dimensional spaces. However, the target in this classification is to design and evaluate its performance other than an accurate estimationObtaining an accurate density estimate non-parametrically is extremely difficult, especially in high-dimensional spaces. The optimal bandwidth kernel is chosen based on several quality parameters and then created PDFs for different lithologies.

After preparing PDFs, the Bayesian technique converted the seismic attributes (PI and V_P_/V_S_) into a lithofacies volume by incorporating non-parametric PDFs with seismic PI & V_P_/V_S_ ratio volume^60^. One classification strategy dealing with complex problems is the Bayesian classification method^38^. It will provide critical knowledge to seismic data classification. The Bayes' rule is essential for statistical data categorization expertise^61^. The Bayes' rule is named a unique reservoir characterization tool due to its combined known classification and prediction classification^62^.

Using Bayes ' theorem, prior knowledge is included in probability estimates^63^. This theorem posits that an event's probability is related to estimating lithofacies and prior probability^64^.

For K number of classes, the Bayes' rule for a class "L" is written,

4 $$p {(}L {|} S) = \frac{{p{(}S {|} L ) p \left( L \right)}}{p\left( S \right)}$$

where $$p \left( S \right) = \mathop \sum \limits_{i = 1}^{k} p {(}S {|} L ) p \left( L \right)$$.

Where:

- *L* is a lithofacies type, i.e., shale or sand
- *S* is a seismic attribute ( a combined attribute of Z_P_ and V_P_/V_S_ ratio)
- *p (L)* is the a priori probability for class *L*.
- *p (S | L)* represents the conditional probability of attributes X knowing we are in class c (for example, distribution of (Z_P_, V_P_/V_S_ ratio) in sand), using the notation for conditional probabilities: "|" means "if."
- *p(S)* is the attributes(S) probability.
- In the prediction of lithology, p (L) is given by the user, and p (S | L) was computed from the PDFs.

## Supplementary Information

Supplementary Information.

## Data Availability

All data generated or analyzed during this study are included in this article.
