# Supplementary material for: Characterization of complex fluvial-deltaic deposits in Northeast India using Poisson impedance inversion and non-parametric statistical technique
Source: Sci Rep. 2022 Oct 8;12:16917. doi: 10.1038/s41598-022-21444-5 (PMC9547880; doi:10.1038/s41598-022-21444-5)
Supplement: Supplementary file 1 — Supplementary Information. [file 41598_2022_21444_MOESM1_ESM.docx]

**Annexure-I**

**Kappa Coefficient Calculation**

**For Conventional classification**

Correctly Classified values (C_i_) = 19,148

Total Number of Values (N_i_) = 30,000

Overall accuracy = Ci/ N_i_ = 19148/30000 = 63.83%

***Kappa Coefficient***

$$\hat{K}=\frac{30000\sum_{1}^{3} (5363+8260+5625)- \sum_{1}^{3} \left( 5545+15386+9069 \right)*10000}{{30000}^{2}-\sum_{1}^{3} \left( 5545+15386+9069 \right)*10000}$$

$$\hat{K}=\frac{274.440.000}{600.000.000}$$

$$\hat{K}=0.4574$$

**For adopted methodology**

Correctly Classified values (C_i_) = 25,013

Total Number of Values (N_i_) = 29,999

Overall accuracy = Ci/ N_i_ = 25,013/29,999 = 83.379%

***Kappa Coefficient***

$$\hat{K}=\frac{29999\sum_{1}^{3} (7843+9398+7772)- \sum_{1}^{3} \left( 8072*10000+9999*13783+8144*10000 \right)}{{29999}^{2}-\sum_{1}^{3} \left( 8072*10000+9999*13783+8144*10000 \right)}$$

$$\hat{K}=\frac{450.388.770}{599.963.784}$$

$$\hat{K}=0.750693$$
